# The nuclear factor E2-related factor 2 and age-related macular degeneration

**DOI:** 10.5935/0004-2749.20230024

**Published:** 2022-02-18

**Authors:** Rogil José de Almeida Torres, Rogério João de Almeida Torres, Andréa Luchini, Ana Lúcia dos Anjos Ferreira

**Affiliations:** 1 Universidade Estadual Paulista “Júlio de Mesquita Filho”, Botucatu, SP, Brazil; 2 Departamento de Oftalmologia, Hospital Angelina Caron, Campina Grande do Sul, PR, Brazil; 3 Centro Oftalmológico de Curitiba, Curitiba, PR, Brazil

**Keywords:** Macular degeneration, NF-E2-Related Factor 2, Oxidation, Antioxidant response element, Enzyme activator, Degeneração macular, Fator nuclear eritroide 2 relacionado ao fator 2, Oxidação, Elemento de resposta antioxidante, Ativador de enzima

## Abstract

After the discovery of anti-vascular endothelial growth factor agents as treatment of wet age-related macular degeneration, the number of studies with the objective to understand the molecular mechanisms involved in the age-re lated macular degeneration genesis has increased. The importance of the nuclear factor e2-related factor 2 lies in its activation-derived proteins being involved in the maintenance of the redox balance and consequent prevention of degenerative macular disease. This article aims to present the characteristics of nuclear factor e2-related factor 2 and describe the main nuclear factor e2-related factor 2-activated antioxidant enzymes that contribute to the preservation of vision.

## Abstract (pt)

Após a descoberta do anti fator de crescimento en dotelial vascular no tratamento da degeneração macular relacionada à idade úmida, muitas pesquisas têm sido realizadas com o intuito de elucidar os mecanismos moleculares envolvidos na gênese da degeneração macular relacionada à idade. O fator nuclear eritroide 2 relacionado ao fator 2 destaca-se pelo fato de diversas proteínas, oriundas de sua ativação, estarem envolvidas na manutenção do equilíbrio do estado redox e consequente prevenção da doença macular degenerativa. Este artigo mostra as características do fator nuclear eritroide 2 relacionado ao fator 2 e descreve as principais enzimas antioxidantes originadas da ativação que contribuem para a preservação da visão.

## INTRODUCTION

Age-related macular degeneration (AMD) is the main cause of irreversible vision loss at old age in developed countries^(1,2)^. Due to the lack of the most efficient alternate preventive measures and/or different therapeutic strategies, it is estimated that AMD prevalence will increase by 40% in 2040^(3)^. AMD pathogenic mechanisms are not thoroughly defined. Risk factors such as age, smoking, environmental and nutritional factors, metabolic dysfunctions, and circulatory, cardiovascular, and genetic disorders make AMD a difficult-to-treat disease. Hence, with the objective to improve the prevention and expand, it is important to fully understand the molecular mechanisms involved in AMD pathogenesis. Transcription factors are proteins responsible for the coordinated expression of genes through specific binding to gene promoter and enhancer sites^(4)^. The Nrf-2 activation induced by the reactive oxygen species (ROS) promotes an increase in the expression of antioxidant enzymes, responsible for maintaining the retinal homeostasis and consequent visual function. A possible association between Nrf-2 deficiency and AMD has been reported^(5)^. In this regard, this study aims to discuss the role of the Nrf-2 in the prevention and/or progression of AMD.

### Nrf-2

**Figure 1:**
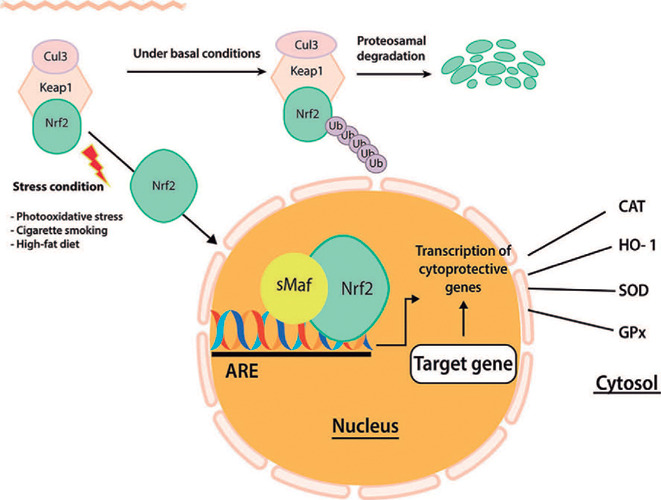
Cellular protection mechanism conferred by Nrf2-ARE pathway.

Nrf-2 was discovered in the 90’s. It is a member of the cap-n-colar family and belongs to a sub-family of basic region leucine zipper (bZip) transcription factors. Nrf-2 is the master antioxidant transcription factor. It induces the expression of over 200 genes that code the proteins and antioxidant enzymes, as well as the phase II metabolizing detoxification enzymes^(6,7,8)^. Hence, Nrf-2 is a critically important mechanism for cell protection and survival^(9,10)^. Importantly, this protection has a cell type-specific target, that is, it modulates gene expression according to each cell type and environment^(11)^. As shown in figure 1, under normal conditions, Nrf-2 is negatively modulated by the kelch-like ECH-associated protein 1 (Keap1), which promotes the degradation of Nrf-2 by the ubiquitin-proteasome pathway. Nevertheless, in situations of oxidative damage associated with pathology, as well as in the presence of chemical compounds with high electrophilic reactivity, such as free radicals, there is a dissociation of the Keap1-Nrf-2 complex with consequent release of Nrf-2 (Figure 1)^(12)^. In this case, the Nrf-2 dissociated from Keap1 translocates into the nucleus, where it recruits small Maf protein (sMaf), forming a heterodimer^(13)^. This heterodimer binds to antioxidant response elements (AREs) or to electrophile response element (EpRE) located in the promoter region of the target genes, initiating transcription^(14)^. Activation of many genes follows, including those that code antioxidant and phase II detoxification enzymes, such as peroxiredoxin 1 (PRX1), nicotinamide adenine dinucleotide phosphate (reduced form; NAD(P)H) quinone oxidoreductase 1 (NQO1), heme oxygenase 1 (HO-1), superoxide dismutase (SOD), catalase (CAT), glutathione S transferases, glutathione reductase (GR), glutathione peroxidase (GPx), thioredoxin (Trx), and glutamate-cysteine ligase (GCL)^(9,15,16)^. They are responsible for the clearance of ROS, providing protection against the accumulation of toxic metabolites^(17)^. It is suggested that Nrf-2 negatively modulates the expression of proinflammatory mediators, including cytokines, adhesion molecules, matrix metalloproteinase 9 (MMP-9), and other inflammatory mediators that affect the activation of nuclear factor-kappa β (NF-κβ), as well as other pathways that control inflammation^(10)^. In this regard, Nrf-2 plays an important role in the protection against several diseases^(18)^. The vicious cycle generated by the unresolved inflammation may be interrupted by the activation of Nrf-2^(10)^. Nrf-2 and NF-κβ pathways interact in a complementary and specialized way for the maintenance of cellular homeostasis^(14)^. However, when chronic inflammatory stimuli persist, activation of NFκB prevails, causing cell death, tissue damage, and fibrosis^(19)^.

### Oxidative stress, infammation, and AMD

The retina, mainly the macular region, where light rays converge, is a tissue exposed to oxidative stress due to high metabolism, large concentrations of polyunsaturated fatty acids, exposure to visible light (between 400-700 nm), and presence of photosensitive molecules, such as rhodopsin and lipofuscin^(20)^. Additionally, retinal pigment epithelial (RPE) cells are rich in mitochondria, producing a large amount of ROS, which are by-products of the respiratory chain activity ^(21)^. The oxidative and nitrosative stress, induced by the imbalance between the antioxidant defense and the production of ROS and reactive nitrogen species (RNS), plays an important role in the triggering and progression of AMD^(20,22)^. Photosensitive reactions, for example, generate ROS and RNS, such as superoxide (O2¯•), hydrogen peroxide (H_2_O_2_), singlet oxygen (1O_2_), and peroxynitrite (ONOO-), which damage RPE cells^(23)^. The hypofunctioning RPE cells inhibit the degradation of the products from the phagocytosis of the photoreceptor outer segment cells, causing the pathological accumulation of lipids in the Bruch’s membrane^(24)^, druses, a hallmark of AMD, and other extracellular deposits in the Bruch’s membrane. These deposits are important risk factors for the development of AMD^(24)^. The drusen contain immunological and inflammatory markers, such as serum amyloid P component (SAP), apolipoprotein E, immunoglobulin light chains, factor X, prothrombin, complement proteins (C3a, C5a, and C5b-9 complex), C-reactive protein (CRP), vitronectin, ubiquitin, and integrins^(25,26)^. Beside drusen, the choriocapillaris, the RPE cells, and photoreceptors also contain inflammatory and immunological markers, such as factor X, fibrinogen, immunoglobulin, HLA-DR, amyloid A component, apolipoprotein B/E, CRP, complement C3, C5, monocyte chemoattractant protein-1 (MCP-1), prothrombin, ubiquitin, and vascular endothelial growth factor (VEGF)^(27,28)^. The intracellular multiprotein complex, inflammasome, also plays an important role in activating the enzymes of the cysteine-aspartic proteases family (caspases). Therefore, the role of the NLR family pyrin domain containing 3 (NLRP3) inflammasome in AMD pathogenesis has been extensively investigated. The drusen present a rich proteinaceous composition, including complement regulators, amyloid-beta (A*β*), and oxidation by-products^(29)^, closely related to the activation of NLRP3 inflammasome^(30,31)^.

### The role of Nrf-2 in the AMD pathogenesis

The sensory retina and the RPE are exposed to high levels of prooxidant and inflammatory stimuli^(20)^. Nevertheless, in young people, the antioxidant machinery in the RPE and sensory retina cells neutralizes the physiologically or pathologically originated ROS^(32)^. The Nrf-2-pathway is a master regulator of stress response in RPE, and it is also a key component of the transduction machinery to maintain proteostasis, which is altered in AMD^(33)^. Besides its antioxidant activity, some studies demonstrated that Nrf-2 is involved in maintaining mitochondria and metabolism, controlling the expression of several tricarboxylic acid cycle (TCA) enzymes, increasing fatty acid oxidation and glycolysis, promoting expression of alcohol dehydrogenase, aldehyde dehydrogenase, or NADPH alenol oxidoreductase, which are involved in the rejuvenation of the mitochondrial function, hence, playing an important role in AMD pathogenesis^(34)^.

Mutations in *Nrf-2* have been associated with a higher risk of AMD development. Identified from DNA extracted from peripheral blood lymphocytes of wet and dry AMD patients, a single mutation of *Nrf-2* at 25129A>C increases the risk for AMD. The C/C genotype showed a predilection for dry AMD whereas an A/C genotype decreased the likelihood of AMD. The C/C genotype was found to be particularly detrimental when linked with age, bad dietary habits, smoking habits, and apparent family history^(35)^. A study suggested that disruption of the *Nfe2l2* gene increased the vulnerability of the outer retina to age-related degeneration. It was observed that *Nrf-2*-deficient mice developed ocular pathology similar to human AMD, and deregulated autophagy is likely a mechanistic link between oxidative injury and inflammation^(36)^. These data strongly suggest that Nrf-2-Keap1 and autophagy together ensure protein quality control and maintain metabolic homeostasis, thereby protecting aging RPE from oxidative stress-induced degeneration. It has been suggested that Nrf-2-pathway impairment contributes to RPE degeneration in AMD and that molecules enhancing Nrf-2 activity may be of interest for this pathology^(37)^. Knockout (KO) animals for *Nrf-2* or its downstream genes (i.e., *HO-1*) develop age-related RPE degeneration and other AMD-like features^(38)^. Another study analyzed the impact of antioxidant enzymes and products of macromolecules oxidative modification on the development of AMD in 308 patients. It was concluded that aging was strongly associated with the oxidative stress and antioxidant status of the tested patients. An inverse relationship of tested oxidant and antioxidant parameters was recorded, and a positive association between the antioxidant enzymes was determined^(39)^. Antioxidant enzymes (SOD, CAT, and GPx) play a vital role in protecting photoreceptors and RPE cells from oxidative damage^(40,41)^. A study reported a negative correlation of patients’ age with the antioxidant enzymes, SOD, and Selenium-dependent GPx and a positive correlation between GR and aging provided that SOD and GPx activities decreased while GR activity rose in AMD patients, especially in the exudative form of the disease^(42)^.

It is known that a variety of antioxidant enzymes and antioxidants are widely distributed in the retina. It seems that each antioxidant has a different role in oxidative stress^(43)^. Figure 1 shows the main Nrf-2-activated antioxidant and phase II detoxifying enzymes that neutralize ROS in the sensory retina and RPE, described below.

### Glutathione Redox cycle and enzymes

This ubiquitous tripeptide L-y-glutamyl-L-cysteinylglycine, or glutathione (GSH), usually the most prevalent intracellular thiol, is known to affect many important biological processes, including the synthesis of proteins and DNA, transport, enzyme activity, metabolism, and cellular protection. The multifunctional properties of this small molecule have raised a growing interest in diverse topics, such as enzyme mechanisms, biosynthesis of macromolecules, intermediary metabolism, drug metabolism, radiation, cancer, oxygen toxicity, transport, immune phenomena, endocrinology, environmental toxins, and aging^(44)^. Glutathione is synthesized intracellularly and can be found in the body in its reduced (GSH) and oxidized (GSSG) forms^(44)^.

Free GSH is mainly present in its reduced form, which may be converted into the GSSG by the GPx during oxidative stress. GSSG may be reverted into its reduced form by GR. Although GR is not directly an antioxidant, its proper function is essential for the maintenance of available reduced GSH, a potent scavenger of the ROS, especially hydrogen peroxide (H_2_O_2_). A high level of GSH may account for at least two processes: an enhanced GSH biosynthesis and a higher conversion of GSSG into GSH by GR. On the other hand, under conditions of marked toxicity or oxidative stress, intracellular GSSG increases substantially^(45)^.

Glutathione, the major water-soluble antioxidant, functions primarily in the cytoplasm and mitochondria. However, the efficiency of the GSH redox system declines with age, predisposing RPE to increased oxidative-stress-mediated damage^(46,47)^. Intracellular GSH levels in the retina are known to also decrease under various pathological conditions, such as diabetic retinopathy^(48)^, glaucoma^(49)^, and retinal photo-oxidative damage^(50)^. Earlier studies demonstrated that GSH depletion could induce apoptosis^(51)^ or necrosis in RPE cells^(52)^, as well as induce ferroptosis, autophagy, and stress-induced premature senescence in RPE cells^(53)^. Ferroptosis, a form of regulated cell death, initiated by lipid peroxidation, is regulated by distinct molecular pathways and was shown to play an important role in degenerative and neoplastic diseases^(54,55^, ^56)^. The hypothesis that AMD may result from oxidative injury to the RPE is supported by pathological studies indicating that damage to the RPE is an early event in AMD and by *in vitro* studies showing that the oxidants induce apoptosis of RPE cells, which are protected by GSH^(57)^. Light impinging on the retina and RPE is a source of oxidative stress, which can induce compensatory upregulation of antioxidant enzymes^(58)^ and can be partially normalized by the effect of GSH and thioredoxin^(50)^. It was suggested that GSH depletion may cause unregulated oxidative stress in retinal cells and increased retinal cell death. The cells in the inner nuclear layer seemed to be affected earlier than the cells in the other layers of the retina^(59)^.

### Glutathione peroxidase

GPx, first described in 1957^(60)^, is present in a number of tissues^(61)^. The GPx enzymatic activity controls hydrogen peroxide and lipid hydroperoxide levels, resulting from an attack of ROS^(44)^. It consists of four apparently identical subunits, which contain one atom of selenium (Se) each^(61)^. It has long been known that Se is an essential nutrient. Se-deficient animals have markedly decreased GSH peroxidase activity^(62)^. GPx labeling is present in the inner retinal layers and RPE but is weak in other layers of the neural retina^(43,63)^. It is enriched in the posterior pole, which is constantly exposed to light but is not enriched in the peripheral retina^(43)^.

The role of GPx in the glutathione redox mechanism is significant; nevertheless, the relationship between GPx plasmatic levels and AMD incidence is inconclusive. A population-based, cross-sectional study on cataracts and AMD and their risk factors, which included 2584 participants, demonstrated that the higher level of GPx plasma was associated with a nine-fold increase in the prevalence of late but not early AMD^(64)^. These results contradict those observed by other authors, who reported a significant reduction in the GPx activity in the AMD group when compared with the control group^(65,66)^.

### Glutathione reductase

GR is an enzyme that, together with the GPx, acts to remove ROS. GR and the glucose-6-phosphate dehydrogenase enzymes are present in the rat retina, rat rod outer segments, and bovine rod outer segments. They are found in a high concentration in the retina and RPE^(67,68^, ^69)^. In relation to AMD, the GR activity was lower in patients with AMD compared with those in the control group^(66,70,71)^.

### Superoxide dismutase

SOD plays a fundamental role in defense against ROS, as it removes superoxide (O2-), forming O_2_ and H_2_O^(72)^. In the eucaryotic systems, there are two forms of SOD. The copper and zinc (CuZn)-SOD is mainly present in the cytosol whereas the manganese-dependent SOD-2 (Mn-SOD) is primarily found in the mitochondria^(73)^. According to Indo, the superoxide generated from mitochondria plays an important role in oxidative stress-related diseases and aging, and that mitochondrial Mn-SOD is an essential antioxidant enzyme for the maintenance of cellular resistance to oxidative stress^(74)^. Lower activities of SOD isoenzymes were reported in tears, cornea, sclera, aqueous humor, and lens while the highest activity was reported in the retina^(75)^. Conversely, Mn-SOD is located in the RPE cells and rod inner segment in the normal rat retina^(76)^. It has been inferred that reduced Mn-SOD levels are associated with AMD progression^(77)^. Previous studies have shown that exposure to light increases phagocytosis of the rod outer segments^(78)^ and produces superoxide anion in RPE cells^(79)^, which is eliminated by SOD. Studies have suggested that epigenetic control of the *Mn-SOD* gene may accelerate AMD progression due to its mitochondrial dysfunction and H_2_O_2_ accumulation, which increase oxidative damage and death of RPE cells^(80,81)^. It was reported that *SOD*-knockout mice are more damaged by light^(82)^, and the lack of CuZn-SOD could accelerate age-related pathological changes in the human retina, such as drusen, thickened Bruch’s membrane, and retinal neovascularization^(83)^. Although the studies conducted *in vitro* coherently indicate the role of SOD in oxidative stress responses, they do not clearly show its association with AMD^(40)^.

### Heme oxygenase 1

Heme is a molecule formed by the protoporphyrin IX which contains an iron atom in its ferric state (Fe2+). It is involved in biological processes, such as oxygen transport (hemoglobin), cellular respiration (cytochromes), signal transduction (guanylate cyclase), and drug detoxification (Cytochromes P450)^(84)^. Despite its physiological importance, the excessive accumulation of free heme (dissociated from proteins) induces oxidative stress and damages lipid membranes and cell organelles^(85)^. In the intracellular environment, the main heme detoxification mechanism is its degradation by the heme oxygenase microsomal enzyme. HO-1, encoded by the *HMOX1* gene, and the closely related heme oxygenase-2 (HO-2), encoded by the *HMOX2 gene*, convert heme, a powerful prooxidant, into biliverdin, which is then changed to bilirubin, a strong antioxidant. Besides biliverdin, this conversion also results in carbon monoxide and iron, which can damage cells^(86)^. HO-1 serves as an inducible 32-kDa protein, which is highly upregulated by oxidative stress that comprises heme, heat shock, hydrogen peroxide, endotoxin, ultraviolet light, and heavy metals, among others^(87,88)^. Induction of HO-1 provides cytoprotective response by exerting inflammatory, antioxidant, and antiapoptotic effects^(89)^. On the other hand, the disturbances in the proper HO-1 level are associated with the pathogenesis of some age-dependent disorders, including AMD^(90)^. Iron is a prooxidant ion, and its accumulation is toxic for the cells. While releasing free iron, HO-1 modulates iron levels, increasing iron efflux from the cells^(91,92)^. Moreover, HO-1 increases levels of ferritin, which binds iron, protecting the cell from oxidative damage^(93)^. AMD may be exacerbated by retinal iron overload and eyes with macular degeneration showed elevated iron levels in the RPE, Bruch’s membrane, and drusen^(94)^. Moreover, the concentration of retinal iron increases with age^(95)^. Additionally, HO-1 and HO-2 levels were found to decrease with age in RPE cells from eyes with neovascular AMD^(41,96)^. From the genetic perspective, HO-1 (*HMOX1*) and HO-2 (*HMOX2*), both downstream targets of Nrf-2, have associated polymorphisms that have been shown to increase the likelihood of AMD in certain individuals^(97)^. A study suggested that the G→C transversion at the 19th position in the *HMOX1* gene (the 19G>C-*HMOX1* polymorphism, rs2071747) and - 42 + 1444 position in the HMOX2 gene (the - 42 +1444A>G-*HMOX2* polymorphism, rs2270363) may be associated with individual susceptibility to AMD^(98)^.

It has been observed that curcumin, a natural compound found in *Curcuma longa,* protects retina-derived 661W cells and RPE-derived ARPE-19 cells from oxidative stress-mediated damage and upregulated expression of phase II enzymes, such as HO-1 and TRX1, that are mediated by the NRF-2 transcription factor. This study suggests that the HO-1 enzyme has a retinal protective role^(99)^. Similarly, a different study reported that overexpression of HO-1 in photoreceptors protected them from subsequent cellular damage caused by intense light exposure^(100)^. Hence, the HO-1 enzyme plays an important role in the homeostasis and the functioning of the sensory retina.

### Catalase

Catalase is a homotetrameric protein (240kDa) present in cells of plants, animals, and aerobic bacteria, with a higher concentration in the erythrocytes and liver^(101)^. Catalase is mainly found in peroxisomes, mitochondria, and the nucleus. The enzyme decomposes H_2_O_2_ into water and molecular oxygen, an extremely important process to prevent the formation of the •OH radical, which is closely associated with the mechanisms of ROS degradation^(102)^. It represents the main enzyme in the elimination of H_2_O_2_. Nevertheless, when there is an excessive increase in its concentration, catalase prevents the accumulation of methemoglobin^(102)^. In the retina, catalase is located within the peroxisomes of the RPE, performing its important role in the prevention of lipid peroxidation and lysosomal enzyme inhibition through the removal of H_2_O_2_ from the phagosome^(103,104)^. A decrease in the catalase activity in both macular and peripheral RPE cells has been reported from the sixth to the ninth decades of human life^(47)^. Corroborating these findings, another study reports a decrease in the catalase immunoreactivity with age in cytoplasm and lysosomes from macular RPE cells of normal eyes and eyes affected by AMD^(41)^. Nevertheless, another study did not establish a correlation between the catalase serum levels and AMD^(105)^.

### Experimental models of Nrf-2 activators

Several experiments have been performed with the objective to identify substances that can activate Nrf-2 and enhance the expression of Nrf-2 target genes in human RPE cells (ARPE-19 cells). The lutein treatment significantly increased the transcription of NQO1 by 1.7 ± 0.1-fold, glutamate-cysteine ligase regulatory subunit (GCLm) by 1.4 ± 0.1-fold, and HO-1 by 1.8 ± 0.3-fold, leading to an enhanced resistance of RPE cells against oxidative damage^(106)^. Other carotenoids can also activate Nrf-2 in the RPE. Astaxanthin led to an increase (2.3-fold) of nuclear Nrf-2 after incubation of ARPE-19 cells with astaxanthin. This carotenoid also induced transcription levels of NQO1 by 1.3-fold of GCLm by 1.9-fold, and of HO-1 by 2-fold^(107)^. Cells treated with astaxanthin and then exposed to blue light light-emitting diode upregulated the Nrf-2-ARE pathway and facilitated the expression of phase II antioxidant enzymes, HO-1 and NQO-1^(108)^. Zeaxanthin was also observed to increase nuclear Nrf-2 in ARPE-19 cells and upregulated Nrf-2 target genes even more strongly. The transcription levels of NQO1 increased by 3.7-fold, of GCLm by 3.2-fold, and of HO-1 by 4.5-fold. The same study also showed that zeaxanthin induces Nrf-2 target genes and GSH levels in rats and reduces the oxidative damage in the retina of these animals^(109)^. Escin, a natural triterpene-saponin, was reported to increase nuclear Nrf-2 levels and induce NQO1 mRNA by 9-fold and HO-1 mRNA by 17-fold after incubation of ARPE-19 cells with 10 µM of escin for 2 hours. Thus, escin is a stronger inducer than lutein^(110)^. Furthermore, different phenolic compounds enhance Nrf-2 target genes in the RPE. For example, treatment of ARPE-19 cells with 50 µM of canolol for 24 hours led to a 1.5-fold induction of HO-1 mRNA^(111)^. Eriodictyol, a flavonoid found in citrus fruits, induced the nuclear translocation of Nrf-2 and increased HO-1 protein levels, NQO1 protein levels, and glutathione after incubating ARPE-19 cells with eriodictyol at a concentration of 0-100 µM for 2-24 hours^(112)^. Glycyrrhizin, a bioactive triterpenoid saponin extracted from a traditional Chinese medicinal herb, glycyrrhiza, increased expression of Nrf-2 and HO-1, playing a protective role in RPE^(113)^. Hispidin, a polyphenol compound isolated from *Phellinus linteus*, markedly enhanced the expression of Nrf-2, HO-1, NQO-1, glutamate-cysteine ligase catalytic subunit, and GCLM in a dose-dependent manner^(114)^. Curcumin induced expression of the HO-1 and protected cells against oxidative stress in cultured human RPE cells^(99,115)^.

Nrf-2 is an important defense mechanism against oxidative stress, a factor likely to trigger several diseases, including AMD. Surprisingly, few experiments have been performed to include this molecule in the preventive treatment of degenerative macular disease. This review study aims to highlight the importance of Nrf-2 activation in the neutralization of oxygen reactive species, which are continuously formed in the macular region. Nrf-2, as well as the main oxidative enzymes, derived from its activation, which act upon the sensory retina and RPE, are likely to become important preventive and therapeutic targets in AMD.
